# Long-Term Correlation between Influenza Vaccination Coverage and Incidence of Influenza-Like Illness in 14 European Countries

**DOI:** 10.1371/journal.pone.0163508

**Published:** 2016-09-29

**Authors:** Ineke T. Spruijt, Marit M. A. de Lange, Frederika Dijkstra, Gé A. Donker, Wim van der Hoek

**Affiliations:** 1 Centre for Infectious Disease Control Netherlands, National Institute for Public Health and the Environment (RIVM), Bilthoven, The Netherlands; 2 NIVEL, Netherlands Institute for Health Services Research, Utrecht, The Netherlands; Public Health Agency of Canada, CANADA

## Abstract

We aimed to examine the long-term correlation between influenza vaccination coverage and the incidence of influenza-like illness (ILI) in the total and elderly populations of European countries for which data was available on at least six consecutive influenza seasons. We graphically visualised vaccination coverage and ILI incidence trends and calculated Spearman rank correlation coefficients. Additionally, we fitted a negative binomial regression model to estimate the change in ILI incidence per percentage point change in vaccination coverage. We found significant negative correlations for the total population of the Netherlands (ρ = -0.60, p-value = 0.003) and for the elderly populations of England (ρ = -0.80, p-value < 0.001) and Germany (ρ = -0.57, p-value = 0.04). However, results were not consistent, and for some countries we observed significant positive correlations. Only for the elderly in England was there a significant decline in incidence rate per percentage point increase in vaccination coverage (incidence rate ratio = 0.93; 95% confidence interval 0.88–0.99). Based on this ecological study it is not possible to provide evidence for a negative correlation between influenza vaccination coverage and ILI incidence. For future, aetiological studies to assess impact of influenza vaccinations on the population, there is a need for high quality data over long periods of time, on proportion of ILI caused by influenza virus infection, on severe outcome measures such as hospitalisation for influenza, and on other factors that potentially affect influenza transmission.

## Introduction

Influenza causes a high annual burden on people and health care systems. The primary influenza prevention measure is vaccination [1,2]. Due to the high mutation ability of the influenza virus, influenza vaccine effectiveness is commonly measured as moderate [3–5], but it is nevertheless estimated to prevent up to 2.1 million influenza-related cases and 37,200 deaths in Europe per year [6]. This suggests that an increase in vaccination coverage would prevent more influenza-related illness and mortality. To maximize health benefits, the WHO and the Council of the European Union have set a target for influenza vaccination coverage of 75% among the elderly population [7].

However, an ongoing discussion among professionals about the effectiveness, safety and necessity of influenza vaccination, especially during the 2009 pandemic, has created doubt among the general public about getting vaccinated against influenza [8,9]. This doubt may have contributed to the declining trend in influenza vaccination coverage in most European countries over the last few years, with speculation as to an increasing trend in influenza-like illness (ILI) [6,10].

Vaccination is primarily intended to prevent complications from influenza virus infection in the elderly and in people of all ages with chronic medical conditions [2]. Unfortunately, most European countries do not have a surveillance system for severe influenza infections. However, an increase in vaccination coverage is expected to have a certain effect on the incidence of ILI, and thereby also on severe influenza. However, little is known about the long-term correlation between vaccination coverage and ILI incidence. Researchers conducting a study in New Zealand found a negative association between increased influenza vaccination coverage and influenza-related mortality in the elderly over a period of 16 years. They also found a significant decrease in ILI incidence. However, they did not associate this decrease in ILI incidence with the increasing vaccine uptake trend [11]. Another study, performed in the Netherlands, found increasing influenza vaccination coverage with significant declining ILI incidence over a 15-year period [12].

There is little data from other European countries about such secular trends. Therefore, in the present exploratory study we examine the long-term trends in influenza vaccination coverage and ILI incidence in 14 European countries, and possible correlation between these two variables.

## Methods

### Study design and data collection

**Fig 1:**
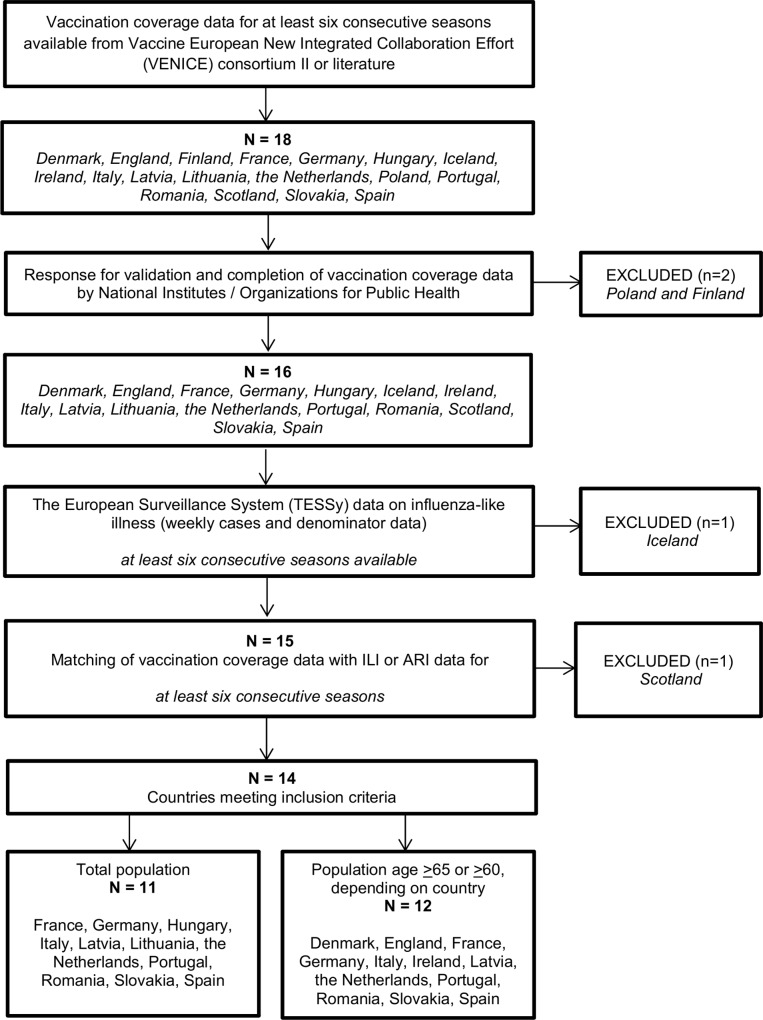
Flowchart of country inclusion and data collection.

We performed an ecological study using country-specific data on the influenza vaccination coverage and ILI incidence for the total population and the elderly population (age ≥65 or ≥60, depending on country). We aimed to study European countries for which data were available for at least six consecutive and matching seasons as to vaccination coverage and ILI incidence. We defined a single influenza season as week 40 of one calendar year through week 20 of the next year. The number of seasons we analysed varied per country due to variation among countries as to their launch of ILI surveillance systems and vaccination monitoring systems. If no ILI surveillance was present in a country, we used data on surveillance of acute respiratory infections (ARI) as a proxy. Fig 1 shows the selection procedure.

#### Vaccination coverage

We investigated vaccination coverage for the total populations and elderly populations in three steps. First, we obtained data on vaccination coverage from the yearly reports of the Vaccine European New Integrated Collaboration Effort (VENICE) consortium, for which data was available from the 2008/2009 through the 2011/2012 season [13–15]. To add data for seasons not covered by VENICE reports, we performed a literature search [16–49]. Finally, to complete coverage data for missing seasons, we approached national influenza vaccination representatives (see acknowledgments). Germany, Hungary, and Slovakia reported vaccination coverage for elderly aged ≥ 60 years, instead of aged ≥ 65 years. For the 2009/2010 season, we collected separate pandemic and seasonal vaccination coverage data. Vaccination coverage data and sources are shown in S1 and S2 Tables.

#### Influenza-like illness

We obtained weekly ILI or ARI data (number of ILI or ARI cases and denominator) from the 1996/1997 season onwards for the total and elderly populations. Weekly data were obtained from The European Surveillance System (TESSy), a European database held by the European Centre for Disease Prevention and Control (ECDC) [50]. Data collection methods are different between countries and are described elsewhere [51]. For the Netherlands, vaccination coverage data was available from the 1991/1992 season onwards. We therefore collected ILI data from the Continuous Morbidity Registration (CMR) of the Netherlands Institute for Health Services Research (NIVEL) [52], as they have ILI data available for seasons prior to 1996/1997.

We checked the weekly ILI or ARI data for missing values and excluded from analysis any season with six or more weeks of missing data. However, before definite exclusion of such a season, we approached national influenza representatives and asked for availability of missing ILI or ARI data. Data from Slovakia (seasons prior to the 2005/2006 season) and Latvia (seasons prior to the 2003/2004 season) were excluded because of differences in the surveillance methods for those earlier seasons (personal communication H. Hudecová of Slovakia (22 September 2014) and R. Nikiforova of Latvia (9 February 2015)). Finally, we calculated seasonal ILI or ARI incidence per 10,000 persons by dividing the sum of weekly ILI or ARI consultations by the mean of the weekly denominator.

ILI and ARI are syndromic diagnoses that are associated with a range of mostly viral pathogens. In routine surveillance systems in Europe only a very small subset of ILI and ARI patients are swabbed for virological examination and data for many countries is incomplete. Specific virological endpoints could therefore not be used in the present analysis. However, we performed a sensitivity analysis using country-specific data on influenza positivity rates. For this sensitivity analyses, we obtained weekly TESSy data on influenza positivity (weekly total number of samples, weekly number of influenza positive samples) from the 1996/1997 season onwards for the total population of each country.

### Statistical analyses

We visualised trends of vaccination coverage and ILI or ARI incidence by using the Spearman rank correlation to assess the strength of the association between them. During exploratory analyses, we noticed that the variance exceeded the mean for each country, indicating overdispersion in the ILI or ARI counts. To account for this overdispersion, we used a negative binomial regression model with log-link function to relate the seasonal ILI or ARI cases to the vaccination coverage [53]. The population (seasonal denominator) was used in the model as an offset. Separate models were fitted for the total population and the elderly persons for each country. The same procedure was used for the sensitivity analysis, using incidence of influenza rather than incidence of ILI or ARI in the analysis. We performed statistical analyses separately for the total population and the elderly persons for each country, using IBM SPSS 21.0.

## Results

Data from 14 European countries were included in this study: 11 countries for the total population and 12 countries for the elderly population. We used ARI data instead of ILI data for Germany, France, and Latvia (Table 1).

**Table 1:** Descriptive statistics and results of analyses for the total population and the elderly population.

Country	Seasons included in analyses	Number of seasons	Median vaccination coverage (%) (min-max)	Change in vaccination coverage (%) ^a^	Median ILI incidence ^b^ (min-max)	Spearman rank correlation		Negative binomial regression model		
						ρ	P-value	IRR ^c^	95% CI	
									Lower	Upper
The total population										
France ^d^	2001/2002–2011/2012	11	21 (18–24)	+3%	5267 (4900–5931)	0.49	0.13	1.00	0.70	1.43
Germany ^d^	2001/2002–2012/2013	12	27 (17–33)	-10%	3579 (3092–4358)	-0.26	0.41	0.99	0.87	1.12
Hungary	2006/2007–2013/2014	8	10 (9–12)	-1%	363 (289–515)	0.87	0.005	1.17	0.58	2.34
Italy	1999/2000–2013/2014	15	18 (11–20)	+5%	884 (413–2164)	0.07	0.81	1.03	0.85	1.27
Latvia ^d^	2003/2004–2013/2014	11	1 (0–14)	-2.2%	3287 (2731–3810)	-0.25	0.47	1.00	0.85	1.17
Lithuania	2005/2006–2012/2013	8	6 (2–8)	+3%	134 (15–162)	-0.04	0.93	1.00	0.68	1.48
The Netherlands	1991/1992–2013/2014	23	18 (7–22)	+12%	172 (79–289)	-0.60	0.003	0.95	0.86	1.04
Portugal	2001/2002–2013/2014	13	17 (14–20)	0%	94 (21–129)	0.10	0.74	1.02	0.73	1.43
Romania	2004/2005–2012/2013	9	8 (3–17)	-4%	9 (5–60)	-0.43	0.24	0.92	0.79	1.07
Slovakia	2006/2007–2013/2014	8	10 (5–13)	+6%	758 (459–1239)	0.75	0.03	1.06	0.85	1.33
Spain	2002/2003–2012/2013	11	23 (14–24)	-5%	208 (146–321)	-0.12	0.72	1.01	0.83	1.22
The elderly (age ≥65 or ≥60, depending on country) population										
Denmark	2002/2003–2013/2014	12	49 (30–55)	+17%	167 (57–310)	-0.11	0.74	0.98	0.90	1.06
England	1996/1997–2012/2013	17	72 (49–75)	+19%	38 (17–143)	-0.81	< 0.001	0.93	0.88	0.99
France ^d^	2001/2002–2013/2014	13	64 (52–67)	-13%	2781 (1896–3443)	0.58	0.04	1.00	0.90	1.12
Germany ^d^	2000/2001–2012/2013	13	49 (31–59) ^e^	+6%	1467 (1128–2105)	-0.57	0.04	0.99	0.92	1.06
Ireland	2003/2004–2013/2014	11	61 (54–70)	-3%	34 (20–54)	0.01	0.99	1.00	0.87	1.15
Italy	1999/2000–2013/2014	15	63 (41–68)	+14%	298 (55–786)	-0.32	0.24	0.98	0.92	1.05
Latvia ^d^	2006/2007–2013/2014	8	2 (2–3)	+0.5%	633 (425–1440)	-0.02	0.95	1.21	0.19	7.77
The Netherlands	1991/1992–2013/2014	23	81 (28–84)	+44%	158 (56–231)	-0.40	0.06	0.99	0.97	1.02
Portugal	1998/1999–2013/2014	16	45 (31–55)	+19%	46 (8–166)	0.09	0.74	0.98	0.91	1.06
Romania	2004/2005–2012/2013	9	19 (15–53)	-2%	3 (2–23)	-0.03	0.93	0.97	0.92	1.02
Slovakia	2006/2007–2013/2014	8	25 (15–36) ^e^	-10%	223 (134–469)	0.57	0.14	1.02	0.93	1.13
Spain	1997/1998–2013/2014	17	64 (56–70)	-11%	61 (32–261)	-0.06	0.82	1.02	0.91	1.15

a Overall change in vaccination coverage trend was calculated taking the difference between the first and last season included in analysis.

b Incidence of influenza-like illness (ILI) or acute respiratory infection (ARI) was calculated by (sum of ILI or ARI cases / denominator (source population))*10,000 persons

c IRR: incidence rate ratio per percentage point change in influenza vaccination coverage

d Country reported acute respiratory infection (ARI), instead of influenza-like illness (ILI)

e Vaccination coverage of the elderly population measured for age ≥60 years instead of ≥65 years

**Fig 2:**
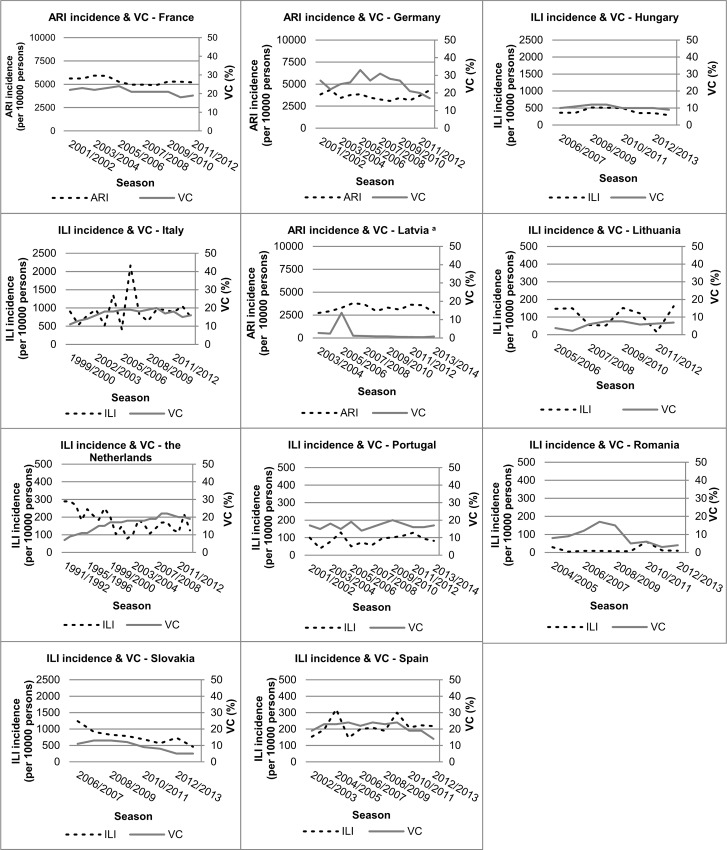
Trends in vaccination coverage (%) and influenza-like illness (ILI) or acute respiratory infection (ARI) incidence (per 10,000 persons) for the total population in European countries. ^a^ Increase in vaccination coverage in 2005/2006 season in Latvia reflects a one-time state funded vaccination campaign (personal communication with R. Nikiforova (27 August 2014).

Visual inspection of the trends for the total population, show initial fluctuating but slightly increasing vaccination coverage trends until the 2008/2009 season in Italy, the Netherlands, Portugal, Romania, Slovakia and Spain (Fig 2). After the 2009 pandemic year, we observe a decline in vaccination coverage in many countries. This is most apparent in Germany, which sustained the highest coverage for the total population (Fig 2, Table 1).

**Fig 3:**
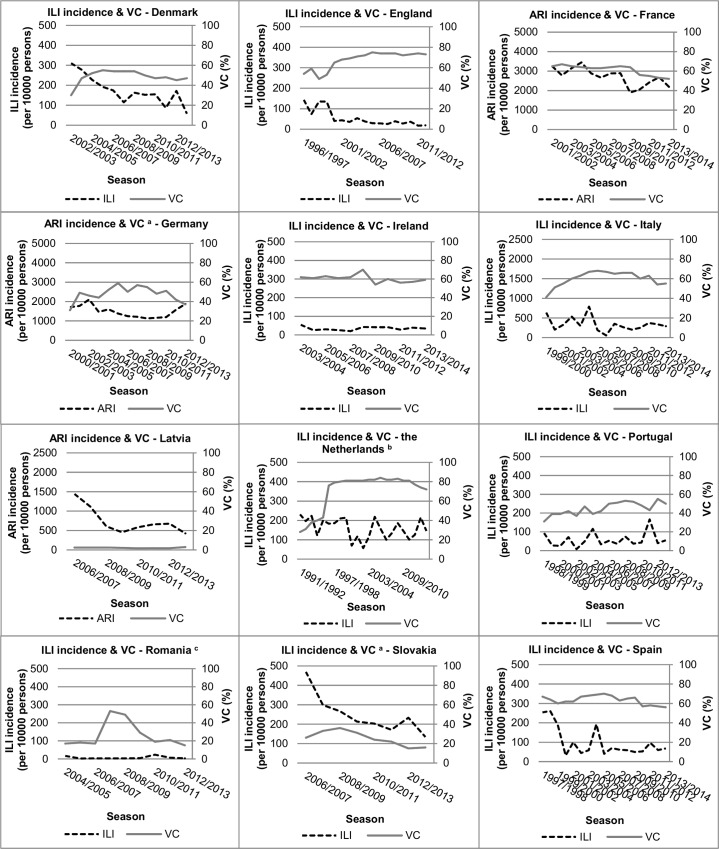
Trends in vaccination coverage (%) and influenza-like illness (ILI) or acute respiratory infection (ARI) incidence (per 10,000 persons) for the elderly population of European countries. ^a^ Vaccination coverage of the elderly population measured for age ≥60 years instead of ≥65 years. ^b^ Increase in vaccination coverage in the 1996/1997 season in the Netherlands was attributable to a new population-wide policy, which reimburses general practitioners for each injected influenza vaccination. ^c^ Increase in vaccination coverage in the 2007/2008 season in Romania was due to active promotion of the influenza vaccine by the National Influenza Centre of Romania (personal communication with V. Alexandrescu (20 February 2015)).

Vaccination coverage trends for the elderly population showed a similar pattern (Fig 3). The Netherlands and England are the only two countries that met or are close to meeting the WHO target of 75% coverage among the elderly.

Changes in vaccination coverage over time were small: changes of at least 5% were measured for Germany, Italy, Slovakia, Spain and the Netherlands. For the elderly population more countries showed changes in vaccination coverage over time of at least 5%.

Three increases were notable in the trends of the vaccination coverage. First, the sharp peak for Latvia in the 2005/2006 season (increase in coverage from 2% to 14%) for the total population is attributable to a one-time state-funded vaccination campaign (personal communication R. Nikiforova (27 August 2014) (Fig 2)). Second, the sharp increase in vaccination coverage in the Netherlands in 1996, especially for the elderly is attributable to a new population-wide policy, which reimburses general practitioners for each injected influenza vaccination (Fig 3). Third, the large increase in the vaccination coverage for the Romanian elderly, from 17% (2006/2007) to 53% (2007/2008), is attributable to active promotion of the influenza vaccine by the National Influenza Centre of Romania (Cantacuzino Institute) (personal communication V. Alexandrescu (20 February 2015) (Fig 3)).

There was a clear declining ILI incidence trend in combination with an increasing vaccination coverage trend for the total population of the Netherlands and for the elderly population of England (Figs 2 and 3). The elderly population of the Netherlands showed a high-sustained vaccination coverage trend from 1996/1997 to 2008/2009 accompanied by a fluctuating ILI incidence trend. England showed an increasing vaccination coverage trend accompanied by a strong declining ILI incidence trend. The elderly population in Denmark, Latvia and Slovakia showed decreasing ILI or ARI incidence trends, but without a clear direction in the vaccination coverage trends. Other countries showed fluctuating trends in vaccination coverage and ILI or ARI incidence for both the total population and the elderly population, without clear direction (Figs 2 and 3).

Significant negative correlation coefficients were found for the total population of the Netherlands (ρ = -0.60, p-value = 0.003) and for the elderly populations of England (ρ = -0.80, p-value = <0.001) and Germany (ρ = -0.57, p-value = 0.04) (Table 1). The correlation coefficient for the Dutch elderly population was marginally significant (ρ = -0.40, p-value = 0.06). We found significant positive correlation coefficients for the total population in Hungary (ρ = 0.87 p-value = 0.005) and Slovakia (ρ = 0.74, p-value 0.03) and for the elderly population in France (ρ = 0.58 p-value = 0.04). A significant decline in ILI incidence per percentage point increase in vaccination coverage was observed only for the elderly population in England (IRR = 0.93, 95%CI = 0.88–0.99). Seasonal data used in all analyses can be found in the S3 Table.

### Sensitivity analyses

For many countries, information on number of swabs taken and proportion of swabs positive for influenza virus, was incomplete, thereby precluding meaningful analysis (S4 Table). A significant negative correlation was only found for France, a country that reports ARI instead of ILI (S5 Table).

## Discussion

We show trends in influenza vaccination coverage and ILI or ARI incidence from 14 European countries. Changes in vaccination coverage over time were small. However, in the majority of the countries vaccination coverage falls for short of the 75% international target and seems to decline in recent years, probably reflecting the negative impact on vaccination uptake of public discussions about safety and benefits of influenza vaccination during 2009 pandemic period.

There were significant negative correlations between influenza vaccination coverage and ILI or ARI incidence in three countries: England and Germany (the elderly population) and the Netherlands (the total population). Only for the elderly population in England did the risk for ILI decrease significantly with each percentage point increase in influenza vaccination coverage. However, the ecological design of the present study and the very different trends in European countries, make it impossible to suggest an association between influenza vaccination coverage and ILI incidence. Significant negative correlations were only observed in countries that have a relatively high vaccination coverage and for which data were available over a long time period. It remains unclear whether the other countries lack the significant negative correlation between vaccination coverage and ILI/ARI incidence, or whether the correlation could not be demonstrated because of short time series, low vaccination coverage or the lack of change in vaccination coverage. Data for more years than are presented here are currently not available. However, the continued routine surveillance of vaccination coverage and ILI incidence in European countries would allow for more robust analysis in future studies.

When interpreting the results of this ecological study, one should take several aspects into account, that are inherent to the differences between countries in structure of their routine surveillance programme and their healthcare system. Some countries report ARI instead of ILI. Median ARI incidence rates are much higher than median ILI incidence rates, as ARI is a less specific diagnosis than ILI. Therefore, the magnitude of ILI and ARI trends cannot be compared among countries as indicators of influenza activity (Table 1). Direct comparison of ILI or ARI trends among countries cannot be made, as definitions have changed over the past 20 years and are still not fully harmonised within Europe. Moreover, the surveillance systems in Europe focus on medically confirmed ILI or ARI, but the proportion of ILI and ARI patients that seek medical care in a country depends on its healthcare and social security systems and on cultural aspects of health-care seeking behaviours [54].

A consequence of using the nonspecific syndromic definition of ILI or ARI as an outcome measure is that it captures not only influenza patients but also patients with other viral or bacterial respiratory infections [55]. With a sensitivity analysis we estimated the number of ILI cases most likely to be due to influenza infection using influenza positivity rates. The outcomes of the sensitivity analyses were not remarkably different from the original analyses, and although information was incomplete for many countries, this suggests that sentinel ILI or ARI data provide a valid proxy for laboratory-confirmed influenza activity [56]. However, it is clear that for meaningful analyses, a continuous systematic sampling approach would be needed.

The large variability in ILI and ARI incidence over time within and between countries, may be related to several other factors. First, a possible decrease in viral fitness over time might have led to a less efficient transmission of the virus between humans, resulting in a declining ILI incidence trend [57]. Second, past influenza outbreaks have likely caused passive immunity among the elderly population, resulting in lower attack rates of influenza [58]. Third, changes in social behaviour like the smaller average family size, improved air quality, and a decrease in smoking might have contributed to the decreased ILI incidence [57]. Fourth, a change in GP consultation behaviour, due to non-reimbursement of drugs like analgesics and cough mixtures, and increased patient knowledge about viral infections may have helped to decrease the number of ILI patients seeking medical care, and thereby decrease the incidence of medically-attended ILI. These factors may explain the declining ILI and ARI incidence trend for the total population of Slovakia and the elderly population of France despite the declining vaccination coverage trend in those countries.

Factors such as seasonal changes in weather, the dominant circulating influenza virus subtypes, and mismatch between vaccine contents and circulating viruses can greatly influence the seasonal magnitude of ILI incidence [59,60,61]. For example, a study in Portugal showed, in general, no significant correlations between vaccination coverage and ILI incidence trends. However, a significant negative linear correlation was found in analyses using only data from influenza A(H3)-dominant seasons [62].

We did not find significant negative correlations between influenza vaccination coverage and ILI incidence for the elderly population in most European countries. However, this does not imply that the elderly do not benefit from influenza vaccination. The main aim of influenza vaccination is prevention of complications from influenza virus infection. A study in Southern Brazil showed a correlation between higher vaccination coverage and lower rates of elderly hospitalisation over a period of 14 years [63]. In a study in Ontario, Canada, higher vaccination coverage was likewise correlated with lower influenza-related hospitalisation rate and lower mortality rate [64]. In France, influenza vaccination also prevented a significant part of influenza-attributable deaths over a 9-year period [65]. These findings support the primary aim of influenza vaccination among the elderly population: to prevent complications from influenza virus infection and influenza-related hospitalisation and mortality [2].

In conclusion, this study shows great heterogeneity between 14 European countries in secular trends of vaccination coverage and ILI or ARI incidence. Although a large amount of routinely collected data was available for this study, it seems that information over more years is required to allow for robust statistical analysis of correlations. Time-series studies on impact of influenza vaccination would requires better information on the proportion of ILI and ARI that is caused by influenza virus infection. In addition, such studies should include information on such variables as dominant influenza virus, vaccine effectiveness, circulation of other respiratory pathogens, and smoking behaviour.

## Supporting Information

S1 Table Influenza vaccination coverage for the total population of European countries. (PDF)

S2 Table Influenza vaccination coverage for the elderly population (age >65 or >60, depending on country) of European countries. (PDF)

S3 Table Seasonal data used in correlation and regression analyses. (XLSX)

S4 Table Overview data ILI samples and percentage influenza positivity for the total population in 11 European countries. (PDF)

S5 Table Sensitivity analyses comparing country-specific data on influenza-positivity with data on influenza-like illness for the total population of European countries. (PDF)
