# Supplementary material for: Long-Term Correlation between Influenza Vaccination Coverage and Incidence of Influenza-Like Illness in 14 European Countries
Source: PLoS One. 2016 Sep 29;11(9):e0163508. doi: 10.1371/journal.pone.0163508 (PMC5042488; doi:10.1371/journal.pone.0163508)
Supplement: S1 Table — (PDF) [file pone.0163508.s001.pdf]

S1 Table. Influenza vaccination coverage for the total population of European countries

Season	Influenza vaccination coverage (percentages)													
	**Denmark**	**England** [a]	**France**	**Germany**	**Hungary**	**Ireland** [a]	**Italy**	**Latvia**	**Lithuania**	**the Netherlands**	**Portugal**	**Romania**	**Slovakia**	**Spain**
1991/1992										7[23]				
1992/1993										9[23]				
1993/1994										10[23]				18[28]
1994/1995										11[23]				
1995/1996										11[23]				18[28]
1996/1997								1[c]		13[23]		2[f]	2[40]	
1997/1998								1[c]		15[23]		2[f]	3[40]	17[28]
1998/1999								2[c]		15[47]	14[37]	2[f]	3[40]	
1999/2000							11[34]	2[c]		17[47]	16[37]	3[f]	5[40]	19[28]
2000/2001							13[34]	2[c]		17[47]		5[f]	6[40]	
2001/2002			22[19]	27[19]	11[b]		14[34]	2[c]		17[47]	17[37]	5[f]	9[40]	
2002/2003			23[19]	22[19]	10[b]		16[34]	2[c]		18[47]	15[37]	5[f]	9[40]	19[19]
2003/2004			22[19]	25[19]	11[b]		18[34]	3[c]		18[49]	18[37]	7[f]	10[40]	23[19]
2004/2005			23[19]	26[19]	12[b]		18[34]	2[c]		18[49]	15[37]	8[f]	11[40]	23[19]
2005/2006			24[19]	33[19]	11[b]		19[34]	14[c, d]	4[e]	18[49]	19[37]	9[f]	11[40]	24[19]
2006/2007			21[22]	27[19]	10[b]		19[34]	1[c]	2[e]	19[49]	14[37]	12[f]	11[40]	22[19]
2007/2008			21[22]	31[21]	11[b]		18[34]	1[c]	6[e]	19[49]	16[37]	17[f]	13[40]	24[20]
2008/2009			21[22]	28[21]	12[b]		19[34]	1[13]	8[e]	22[48]	18[37]	15[f]	13[13]	23[29]
2009/2010	10[46]		21[13]	27[13]	12[b]		20[34]	1[13]	8[e]	22[48]	20[37]	5[13]	12[13]	24[29]
2010/2011	10[46]		18[22]	21[42]	10[14]		17[14]	1[14]	6[e]	21[48]	18[14]	6[14]	9[14]	19[29]
2011/2012	10[46]		19[22]	20[42]	10[15]		18[15]	1[c]	6[15]	20[48]	16[15]	3[15]	8[15]	19[29]
2012/2013	10[46]			17[42]	10[b]		15[34]	0.4[c]	7[e]	20[48]	16[47]	4[f]	5[40]	14[27]
2013/2014	11[46]				9[b]		16[34]	1[c]		19[48]	17[35]		5[40]	

[a] England and Ireland do not monitor influenza vaccination coverage in the total population
[b] Personal communication with Dr. Z. Mòlnar (30 May 2014)
[c] Personal communication with Dr. R. Nikiforova (27 August 2014)
[d] State-funded vaccination campaign (personal communication with Dr. R. Nikiforova (27 August 2014))
[e] Personal communication with Dr. E. Orechoviene (27 August 2014)
[f] Personal communication with Dr. H. Hudecová (26 May 2014)
