# Supplementary material for: Long-Term Correlation between Influenza Vaccination Coverage and Incidence of Influenza-Like Illness in 14 European Countries
Source: PLoS One. 2016 Sep 29;11(9):e0163508. doi: 10.1371/journal.pone.0163508 (PMC5042488; doi:10.1371/journal.pone.0163508)
Supplement: S2 Table — (PDF) [file pone.0163508.s002.pdf]

S2 Table. Influenza vaccination coverage for the elderly population (age ≥65 or ≥60, depending on country) of European countries

Season	Influenza vaccination coverage (percentages)													
	**Denmark**	**England**	**France**	**Germany** [a]	**Hungary** [a]	**Ireland**	**Italy**	**Latvia**	**Lithuania**	**the Netherlands**	**Portugal**	**Romania**	**Slovakia** [a]	**Spain**
1990/1991		42[30]					18[43]							
1991/1992		46[30]					22[43]			28[23]				
1992/1993		46[30]					26[43]			31[23]				
1993/1994		52[30]					29[43]			38[23]				
1994/1995		54[30]					32[43]			40[23]				
1995/1996		55[30]					35[43]			43[23]				
1996/1997		54[30]					38[43]			76[23]		8 [f]		63[33]
1997/1998		59[30]					41[43]			79[25]		10 [f]		67[33]
1998/1999		49[30]					49[43]			80[47]	31[37]	15 [f]		64[33]
1999/2000		53[30]					41[34]			81[47]	39[37]	15 [f]		60[33]
2000/2001	46[31]	65[17]	65[36]	31[31]	31 [b]		51[34]			81[47]	39[31]	17 [f]	21[36]	62[32]
2001/2002		68[17]	65[36]	49[24]	33 [b]		55[34]			81[47]	42[37]	17 [f]	32[36]	62[32]
2002/2003	30[45]	69[17]	67[36]	46[24]	37 [b]		60[34]			81[47]	37[37]	18 [f]		67[32]
2003/2004	47[45]	71[17]	65[36]	44[24]	40 [b]	62[36]	63[34]			82[49]	47[37]	19 [f]	38[36]	68[32]
2004/2005	52[45]	72[17]	64[19]	52[24]	39 [b]	61[36]	67[34]			82[49]	39[37]	17 [f]	23[36]	69[32]
2005/2006	55[45]	75[17]	63[19]	59[24]	38 [b]	63[36]	68[34]		2 [e]	84[49]	42[37]	18 [f]	29[36]	70[32]
2006/2007	54[45]	74[17]	63[19]	50[19]	34 [b]	61[36]	67[34]	2 [d]	2 [e]	82[49]	50[37]	17 [f]	26[36]	68[32]
2007/2008	54[45]	74[17]	64[36]	57[21]	35 [b]	62[36]	65[34]	2 [d]	13 [e]	82[49]	51[37]	53 [f,g]	33[36]	63[32]
2008/2009	54[13]	74[13]	65[26]	55[13]	38[13]	70[13]	66[34]	2[13]	24 [e]	83[48]	53[37]	49[13]	36[36]	65[32]
2009/2010	50[43]	72[13]	64[26]	48[13]	32[13]	54[13]	66[34]	2[13]	22 [e]	81[48]	52[37]	29[13]	31[36]	66[13]
2010/2011	47[43]	73[14]	56[26]	51[14]	30[14]	60[14]	60[34]	2[14]	17 [e]	81[48]	48[37]	19[14]	24[36]	57[14]
2011/2012	48[43]	74[15]	55[26]	42[42]	31[15]	56[15]	63[34]	2[15]	19[15]	77[48]	43[15]	21[15]	22[36]	58[15]
2012/2013	45[43]	73[17]	53[26]	37[42]	31 [b]	57 [c]	54[34]	2 [d]	19 [e]	74[48]	55[44]	15 [f]	15[39]	57[32]
2013/2014	47[43]	73[18]	52[26]		29 [b]	59(27)	55[34]	3 [d]		72[48]	50[35]		16[40]	56[32]
2014/2015	45[43]	73[41]	48[26]		27 [b]								14[38]	56[32]

[a] Vaccination coverage of the elderly population measured for age ≥60 years instead of age ≥65 years

[b] Personal communication with Dr. Z.Mòlnar (30 May 2014)

[c] Personal communication with Dr. S. Cotter (26 May 2014)

[d] Personal communication with Dr. R. Nikiforova (27 August 2014)

[e] Personal communication with Dr. E. Orechoviene (27 August 2014)

[f] Personal communication with Dr. H. Hudecová (26 May 2014)

[g] Active promotion of the influenza vaccine by National Influenza Centre of Romania (personal communication with Dr. V. Alexandrescu (20 February 2015))
