# Supplementary material for: Long-Term Correlation between Influenza Vaccination Coverage and Incidence of Influenza-Like Illness in 14 European Countries
Source: PLoS One. 2016 Sep 29;11(9):e0163508. doi: 10.1371/journal.pone.0163508 (PMC5042488; doi:10.1371/journal.pone.0163508)
Supplement: S4 Table — (PDF) [file pone.0163508.s004.pdf]

S4 Table. Overview data ILI samples and percentage influenza positivity for the total population in 11 European countries

Season		Germany	France	Hungary	Italy	Latvia	Lithuania	The Netherlands	Portugal	Romania	Slovakia	Spain
1996/1997	% Influenza positive [a]							28.0				
	∑ Samples							454				
	∑ Influenza cases							127				
	No samples reported							1				
	Missing weeks							4				
	Total missing							5				
1997/1998	% Influenza positive [a]							24.2				
	∑ Samples							343				
	∑ Influenza cases							83				
	No samples reported							1				
	Missing weeks							-				
	Total missing							1				
1998/1999	% Influenza positive [a]							24.4				
	∑ Samples							414				
	∑ Influenza cases							101				
	No samples reported							-				
	Missing weeks							3				
	Total missing							3				
1999/2000	% Influenza positive [a]				22.8			28.5				
	∑ Samples				988			284				
	∑ Influenza cases				225			81				
	No samples reported				-			3				
	Missing weeks				18			-				
	Total missing				18			3				
2000/2001	% Influenza positive [a]				13.6			21.2				
	∑ Samples				81			66				
	∑ Influenza cases				11			14				
	No samples reported				6			7				
	Missing weeks				11			1				

	Total missing				17			8				
2001/2002	% Influenza positive [a]	24.3	25.8		20.8			61.8	57.7			
	∑ Samples	3215	3658		48			55	246			
	∑ Influenza cases	780	944		10			34	142			
	No samples reported	5	-		5			6	4			
	Missing weeks	-	5		11			10	7			
	Total missing	5	5		16			16	11			
2002/2003	% Influenza positive [a]	46.4	18.8		16.1			26.8	24.3			25.1
	∑ Samples	4623	4404		56			56	152			1302
	∑ Influenza cases	2145	827		9			15	37			327
	No samples reported	2	-		10			8	2			1
	Missing weeks	6	2		8			-	1			1
	Total missing	8	2		18			8	3			2
2003/2004	% Influenza positive [a]	19.9	28.1		13.7	46.7		30.4	57.4			24.0
	∑ Samples	2785	3878		2882	105		112	272			1264
	∑ Influenza cases	553	1091		395	49		34	156			303
	No samples reported	3	-		-	19		6	4			
	Missing weeks	1	2		9	3		-	-			
	Total missing	4	2		9	22		6	4			
2004/2005	% Influenza positive [a]	31.5	27.9		30.9	17.1		44.3	54.4	19.7		41.3
	∑ Samples	4217	4836		2840	88		158	349	854		1696
	∑ Influenza cases	1329	1351		878	55		70	190	168		701
	No samples reported	-	-		1	9		9	6	5		
	Missing weeks	4	3		8	-		-	-	1		
	Total missing	4	3		9	9		9	6	6		
2005/2006	% Influenza positive [a]	32.5	18.6		9.6	39.7	13.3	32.4	27.0	10.1		32.3
	∑ Samples	2041	4427		2279	63	83	306	159	755		1401
	∑ Influenza cases	663	824		218	25	11	99	43	76		452
	No samples reported	-	1		2	13	20	3	3	1		
	Missing weeks	1	2		6	1	3		-	1		
	Total missing	1	3		8	14	23	3	3	2		
2006/2007	% Influenza positive [a]	45.9	22.5	6.8	37.7	55.1	12.3	24.3	47.3	26.8	36.4	44.8
	∑ Samples	2849	4192	293	1333	69	155	301	188	795	206	1855
	∑ Influenza cases	1307	943	20	503	38	19	73	89	213	75	825

	No samples reported	-	-	1	4	19	19	1	6	6	10	
	Missing weeks	-	3	3	5	1	-		-	-	2	
								1				
	Total missing	-	3	4	9	20	19		6	6	12	
2007/2008	% Influenza positive [a]	45.1	27.4	33.6	14.9	62.5	23.4	29.6	45.7	46.0	50.5	49.1
	∑ Samples	2492	5019	432	859	24	111	483	116	1011	220	2019
	∑ Influenza cases	1125	1376	145	128	15	26	143	53	465	111	992
	No samples reported	-	-	1	4	17	12		5	2	3	
	Missing weeks	-	-	-	9	7	-		-	5	-	
	Total missing	-	-	1	13	24	12		5	7	3	
2008/2009	% Influenza positive [a]	51.7	34.1	20.3	40.8	51.2	38.2	30.1	48.0	36.9	40.5	42.9
	∑ Samples	3277	4799	1017	1284	82	136	568	319	1156	227	2507
	∑ Influenza cases	1694	1638	206	524	42	52	171	153	427	92	1075
	No samples reported	-	1	-	5	17	11		1	4	7	
	Missing weeks	-	2	-	6	-	-		1	-	-	
	Total missing	-	3	-	11	17	11		2	4	7	
2009/2010	% Influenza positive [a]	38.2	35.3	20.7	23.1	23.9	52.0	30.7	38.8	34.6	24.3	45.4
	∑ Samples	2315	8949	1850	744	67	221	925	366	1805	111	8179
	∑ Influenza cases	885	3155	382	172	16	115	284	142	624	27	3714
	No samples reported	-	-	-	6	27	2		8	3	6	
	Missing weeks	2	-	-	2	-	4		-	-	7	
	Total missing	-	-	-	8	27	6		8	3	13	
2010/2011	% Influenza positive [a]	49.5	36.4	13.9	37.5	50.5	44.6	40.0	39.7	28.2	41.7	44.9
	∑ Samples	2810	5295	1948	1524	101	269	633	136	893	187	5482
	∑ Influenza cases	1391	1929	270	571	51	120	253	54	252	78	2463
	No samples reported	-	-	1	-	24	9		10		4	
	Missing weeks	-	-	-	9	-	-				2	
	Total missing	-	-	1	9	24	9				6	
2011/2012	% Influenza positive [a]	23.3	34.7	29.9	49.8	45.2	28.9	19.4	31.6	27.4	33.3	50.0
	∑ Samples	1692	4829	1053	1579	62	104	387	155	668	135	5858
	∑ Influenza cases	394	1676	315	786	28	30	75	49	183	45	2928
	No samples reported	-	-	1	1	20	11		10		6	
	Missing weeks	-	-	-	9	-	-		-		1	
	Total missing	-	-	1	10	20	11		10		7	
2012/2013	% Influenza positive [a]	47.5		28.7	54.2	58.5	71.7	33.2	34.6	27.8	42.1	51.4

	∑ Samples	3868		696	1296	65	537	695	497	454	278	5173
	∑ Influenza cases	1836		200	702	38	385	231	172	126	117	2660
	No samples reported	-		4	1	19	2		2	3	9	
	Missing weeks	-		1	9	-	-		-	-	1	
	Total missing	-		5	10	19	2		2	3	10	
2013/2014	% Influenza positive [a]			15.4	30.2	75.0		15.0	53.0		21.7	
	∑ Samples			663	985	4		340	249		120	
	∑ Influenza cases			102	297	3		51	132		26	
	No samples reported			4	-	29			10		6	
	Missing weeks			2	9	-			-		1	
	Total missing			6	9	29			10		7	

[a] % Influenza positivity = (∑ Influenza cases / ∑ Specimen) * 100
