# Supplementary material for: Long-Term Correlation between Influenza Vaccination Coverage and Incidence of Influenza-Like Illness in 14 European Countries
Source: PLoS One. 2016 Sep 29;11(9):e0163508. doi: 10.1371/journal.pone.0163508 (PMC5042488; doi:10.1371/journal.pone.0163508)
Supplement: S5 Table — (PDF) [file pone.0163508.s005.pdf]

S5 Table. Sensitivity analyses comparing country-specific data on influenza-positivity with data on influenza-like illness for the total population of European countries

Country	Influenza season	ILI cases					Influenza cases				
		Spearman rank		Negative binomial regression model			Spearman rank		Negative binomial regression model		
		Correlation coefficient	p-value	IRR	95%CI		Correlation coefficient	p-value	IRR	95%CI	
					Lower	Upper				Lower	Upper
France	2001/2002 – 2011/2012	0.49	0.13	1.00	0.70	1.43	**-0.73**	**0.01**	0.90	0.63	1.30
Germany	2001/2002 – 2012/2013	-0.26	0.41	0.99	0.87	1.12	-0.09	0.78	0.98	0.87	1.11
Hungary	2006/2007 – 2013/2014	0.87	0.005	1.17	0.58	2.34	0.49	0.22	1.07	0.49	2.34
Italy	1999/2000 - 2013/2014	0.07	0.81	1.03	0.85	1.27	0.09	0.45	1.07	0.87	1.32
Latvia	2003/2004 – 2013/2014	-0.25	0.47	1.00	0.85	1.17	-0.39	0.24	0.98	0.84	1.14
Lithuania	2005/2006 – 2012/2013	-0.04	0.93	1.00	0.68	1.48	0.43	0.29	1.34	0.91	1.98
the Netherlands [a]	1996/1997 – 2013/2014	**-0.60**	**0.003**	0.96	0.80	1.16	-0.20	0.42	0.98	0.81	1.18
Portugal	2001/2002 – 2013/2014	0.10	0.74	1.02	0.73	1.43	0.01	0.96	1.04	0.71	1.54
Romania	2004/2005 – 2012/2013	-0.22	0.54	0.92	0.79	1.07	-0.15	0.70	0.95	0.83	1.10
Slovakia	2006/2007 – 2013/2014	0.74	0.03	1.06	0.85	1.33	0.65	0.08	1.08	0.87	1.36
Spain	2002/2003 – 2012/2013	-0.12	0.72	1.01	0.83	1.22	0.05	0.89	0.99	0.82	1.20

[a] The amount of influenza seasons for the total population used in the sensitivity analyses differs from the amount used in the primary analyses, as data on influenza positivity was only available from the 1996/1997 season onwards.
